# A DAS-Based Multi-Sensor Fusion Framework for Feature Extraction and Quantitative Blockage Monitoring in Coal Gangue Slurry Pipelines

**DOI:** 10.3390/s26072048

**Published:** 2026-03-25

**Authors:** Chenyang Ma, Jing Chai, Dingding Zhang, Lei Zhu, Zhi Li

**Affiliations:** 1 Energy and Mining Engineering College, Xi’an University of Science and Technology, Xi’an 710054, China; chaij@xust.edu.cn (J.C.); zhangdd@xust.edu.cn (D.Z.); lizhi@stu.xust.edu.cn (Z.L.); 2 Key Laboratory of Western Mine Exploitation and Hazard Prevention, Ministry of Education, Xi’an University of Science and Technology, Xi’an 710054, China; 3 China Coal Energy Research Institute Co., Ltd., Xi’an 710054, China; zhulei@chinacoal.com

**Keywords:** distributed acoustic sensing (DAS), coal gangue slurry pipeline, blockage identification, quantitative characterization, multi-sensor collaborative monitoring

## Abstract

**Highlights:**

**What are the main findings?**

- Three blockage-correlated characteristic frequencies (1.5 Hz, 26 Hz, 174 Hz) were identified and cross-validated by synchronous DAS, FBG, and accelerometer data. The 26 Hz component was theoretically verified as the structural natural frequency of the local valve–pipe assembly (Euler–Bernoulli beam model, 25.7 Hz) with a maximum measurement deviation ≤ 0.4 Hz, forming a hierarchical feature perception system for blockage monitoring.
- The DAS phase change rate exhibited a unimodal nonlinear response to blockage degree (first increasing and then decreasing), with the peak response occurring at 40.94% blockage, which was defined as the fluid–structure interaction resonance window for blockage-induced vibration.
- A sine-fitting quantitative inversion model for blockage degree was developed based on the DAS phase change rate, achieving a high goodness of fit (R^2^ = 0.985). Leave-one-out cross-validation confirmed the model’s excellent robustness, with a mean relative prediction error of 3.77% (<5%), realizing the transition from qualitative blockage detection to quantitative severity assessment.

**What are the implications of the main findings?**

- The proposed feature-level fusion framework breaks through the limitations of single DAS monitoring in fixed-point quantitative accuracy and high-frequency sensitivity, offering a novel “distributed + point-type” collaborative monitoring paradigm for long-distance pipeline health monitoring.
- The established hierarchical feature system and quantitative inversion model provide a complete sensor-level solution for real-time early warning, precise localization, and quantitative diagnosis of slurry pipeline blockages. The low-complexity workflow is suitable for field engineering implementation, which is of great significance for ensuring the safe and efficient operation of coal mine green backfill systems.

**Abstract:**

Long-distance coal gangue slurry transportation pipelines are critical components of underground coal mine green backfilling systems, yet blockage failures severely threaten their safe and efficient operation. Existing distributed acoustic sensing (DAS)-based monitoring methods for such pipelines suffer from three key limitations: insufficient fixed-point quantitative accuracy, lack of verified blockage-specific characteristic indicators, and limited quantitative severity assessment capability. To address these gaps, this paper proposes a novel feature-level fusion monitoring method integrating DAS, fiber Bragg grating (FBG), and piezoelectric accelerometers for accurate blockage identification and quantitative evaluation in coal gangue slurry pipelines. A slurry pipeline circulation test platform with gradient blockage simulation (0% to 76.42%) and a synchronous multi-sensor monitoring system were developed. Through multi-domain signal analysis, three blockage-correlated characteristic frequencies were identified and cross-validated by synchronous multi-sensor data: 1.5 Hz (system background vibration), 26 Hz (blockage-induced fluid–structure resonance, verified by the Euler–Bernoulli beam theory with a theoretical value of 25.7 Hz), and 174 Hz (transient flow impact). The DAS phase change rate exhibited a unimodal nonlinear response to blockage degree, with the peak occurring at 40.94% blockage. On this basis, a sine-fitting quantitative inversion model was developed, achieving a high goodness of fit (R^2^ = 0.985), and leave-one-out cross-validation confirmed its excellent robustness with a mean relative prediction error of 3.77%. Finally, a collaborative monitoring framework was built to fully leverage the complementary advantages of each sensor, realizing full-process blockage monitoring covering global blockage localization, precise quantitative severity calibration, and high-frequency transient risk early warning. The proposed method provides a robust experimental and technical foundation for real-time early warning, precise localization, and quantitative diagnosis of long-distance slurry pipeline blockages and holds important engineering application value for the safe and efficient operation of underground coal mine green backfilling systems.

## 1. Introduction

With the development of green coal mining, coal gangue slurry pipeline backfilling has become a key technology for goaf treatment and solid waste disposal [1,2,3,4]. As the core transmission carrier, long-distance pipelines directly affect the operation of backfilling systems. However, pipelines are prone to silting and blockage due to unfavorable slurry properties, pressure fluctuations, and structural changes, which has become the main failure mode threatening mine safety [5,6,7]. Therefore, developing real-time, quantitative blockage monitoring technologies for long-distance, high-interference pipelines is of urgent engineering significance.

Traditional monitoring methods (acoustic emission, infrared thermal imaging, and pressure monitoring) have inherent limitations in monitoring distance, anti-interference performance, and positioning accuracy [8,9,10]. Distributed acoustic sensing (DAS) offers distributed, continuous monitoring with high sensitivity and immunity to electromagnetic interference, demonstrating significant potential for long-distance pipeline safety monitoring applications [11,12]. At present, research and application of DAS technology are primarily focused on the detection and localization of oil and gas pipeline leaks [13,14,15]. Scholars have achieved qualitative identification and spatial localization of leak events by optimizing optical fiber layout and time–frequency analysis methods [16,17,18]; some studies have extended this technology to the monitoring of flow field parameters such as fluid velocity and solid content and initially revealed the correlation between flow-induced vibration and DAS response signals [19,20,21].

While multi-sensor data fusion methods have been proven effective in improving pipeline leak localization accuracy, and identification technologies based on DAS and multi-dimensional information fusion have made progress in pipeline external threat monitoring [22,23], existing studies still have clear limitations in monitoring the “internal interception” blockage of slurry pipelines, which can be summarized as follows: (1) most studies target oil and gas “leakage faults” and ignore the unique fluid–structure interaction vibration characteristics of slurry “internal blockage” faults [13,14,15]; (2) blockage monitoring remains at the level of qualitative abnormality identification, lacking a verified characteristic frequency system and quantitative characterization model; (3) standalone DAS technology suffers from insufficient fixed-point quantitative accuracy and high-frequency sensitivity, and no clear feature-level fusion method for heterogeneous multi-sensor data has been proposed [24,25].

To address these gaps, this study develops a DAS-based multi-sensor fusion framework for quantitative blockage assessment in coal gangue slurry pipelines—a capability rarely to be realized in existing DAS monitoring approaches. A slurry pipeline circulation test platform with gradient blockage simulation (0% to 76.42%) and a synchronous multi-sensor monitoring system were first established. The three core contributions of this study are summarized below:

(1) Three blockage-correlated characteristic frequencies (1.5 Hz, 26 Hz, 174 Hz) are identified and cross-validated by synchronous multi-sensor data, with the 26 Hz component theoretically verified as the structural natural frequency of the local valve–pipe assembly via the Euler–Bernoulli beam theory (theoretical value: 25.7 Hz), establishing a hierarchical feature perception system for slurry pipeline blockage monitoring.
(2) A sine-fitting quantitative inversion model based on the DAS phase change rate is developed, achieving a high goodness of fit (R^2^ = 0.985) and realizing the quantitative evaluation of pipeline blockage severity.
(3) A novel “distributed + point-type” feature-level fusion framework is proposed, which fully leverages the complementary advantages of multi-sensors, compensates for the inherent limitations of standalone DAS monitoring, and provides a referable engineering paradigm for long-distance pipeline health monitoring.

These contributions collectively lay a robust theoretical and experimental foundation for real-time early warning, precise localization, and quantitative diagnosis of blockages in long-distance coal gangue slurry pipelines.

## 2. Theoretical Principle of DAS and Multi-Sensor Collaborative Perception

**Figure 1:**
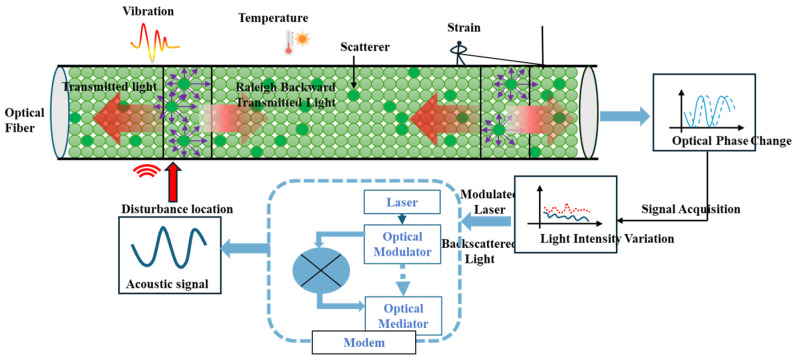
Schematic diagram of the DAS measurement principle (adapted from Ref. [29]).

DAS is a vibration monitoring technology based on the Rayleigh backscattering effect in optical fibers. Its core principle is to use a single-mode optical fiber as both the sensing medium and the signal transmission carrier, enabling large-scale distributed vibration monitoring [26]. When light waves propagate in the fiber core, they interact with environmental factors around the optical fiber, such as vibration and strain. These interactions induce slight changes in the intensity, frequency, and phase of the optical signal. A typical DAS system mainly consists of two core parts: a demodulator (modem) and a sensing optical fiber. The laser inside the demodulator emits light, which is modulated by the optical modulator and injected into the sensing fiber. During propagation in the fiber core, the light generates Rayleigh backscattered light, which carries the optical change information induced by external vibration and returns to the demodulator along the original path. Through demodulation and analysis of the Rayleigh backscattered signal, the tiny changes in light phase and intensity can be converted into the corresponding vibration signal, thus realizing real-time monitoring of vibration along the optical fiber [27,28], as shown in Figure 1.

Optical phase modulation is a core physical basis for optical fiber sensing and a key enabler for DAS technology to achieve high-sensitivity vibration and strain monitoring. When the optical fiber is subjected to axial external stress, it simultaneously induces changes in the physical length of the sensing fiber and the effective refractive index of the fiber core, which in turn leads to a change in the optical path difference in the transmitted light wave and ultimately causes a phase shift of the optical signal. The optical fiber phase change Δϕ is mainly induced by the fiber length change $\Delta L_{f}$ and the refractive index change Δn caused by the photoelastic effect [30]:

(1) $$\Delta \emptyset =\frac{2\pi n}{\lambda }\Delta L_{f}+\frac{2\pi L_{f}}{\lambda }\Delta n=\frac{2\pi n}{\lambda }\Delta L_{f}\left(\varepsilon +\frac{\Delta n}{n}\right)$$

where Δ∅ is the phase of the backscattered light (rad); *n* is the refractive index of the optical fiber; *λ* is the wavelength of the laser pulse (m); *L_f_* is the effective propagation length of the optical fiber (in meters, m); $\varepsilon =\Delta L/L_{f}$ is the axial strain (*με*).

When the optical fiber is subjected to axial strain ε, it undergoes lateral contraction strains $\varepsilon_{xx}=\varepsilon_{yy}=-\mu \varepsilon$. When considering the coupling of lateral and longitudinal strains, the relationship between the photoelastic effect-induced refractive index change Δn and the axial strain ε is expressed as:

(2) $$\Delta n=-\frac{n^{3}}{2}\left[\left(1-\mu \right)P_{12}-\mu P_{11}\right]\varepsilon$$

where *P*_11_ and *P*_12_ are photoelastic coefficients; *μ* is Poisson’s ratio of the optical fiber.

Substituting Equation (2) into Equation (1) yields the relationship between the phase change monitored by DAS and the axial strain of the optical fiber as:

(3) $$\Delta \emptyset =\frac{2\pi nL_{f}}{\lambda }\left[1-\frac{n^{2}}{2}\left(1-\mu \right)P_{12}-\mu P_{11}\right]\varepsilon =K\varepsilon$$

where $K=\frac{2\pi nL_{f}}{\lambda }\left[1-\frac{n^{2}}{2}\left(1-\mu \right)P_{12}-\mu P_{11}\right]$, which is determined by the intrinsic properties of the optical fiber.

Coal gangue slurry is a typical two-phase solid–liquid flow [31]. Pipeline blockage reduces the flow cross-sectional area, causing local flow field disturbances and fluid excitation, which, in turn, excite the pipeline structure to produce flow-induced vibration. According to the basic equations of structural dynamics, there is a quantitative correlation between the blockage-induced fluid excitation force and the pipeline vibration acceleration:

(4) $$F(t)=m\cdot \frac{d^{2}u(t)}{dt^{2}}$$

where F(t) is the fluid excitation force induced by blockage (N); m is the mass per unit length of the pipeline (kg/m); u(t) is the lateral vibration displacement of the pipeline (m); $d^{2}u(t)/dt^{2}$ is the pipeline vibration acceleration (m/s^2^).

The pipeline vibration displacement is coupled with the surface structural strain under the constraint of structural mechanics. The surface axial strain of the circular slurry pipeline caused by vibration satisfies the following relationship:

(5) $$\varepsilon_{p}(t)=\frac{D_{i}}{2}\cdot \frac{d^{2}u(t)}{dx^{2}}$$

where $\varepsilon_{p}(t)$ is the surface axial strain of the pipeline (*με*); $D_{i}$ is the outer diameter of the pipeline (m); $d^{2}u(t)/dx^{2}$ is the vibration displacement curvature of the pipeline.

The strain generated by pipeline structure vibration is transmitted to the laid sensing optical fiber via the binding contact interface, enabling strain coupling between the pipeline and the optical fiber. Based on the strain transfer theory, the coupling process is linearly correlated [32]:

(6) $$\varepsilon_{f}(t)=\eta \cdot \varepsilon_{p}(t)$$

where $\varepsilon_{f}(t)$ is the axial strain of the sensing optical fiber (*με*); *η* is the pipeline–fiber strain transfer coefficient, which is determined by the optical fiber laying mode, binding process, and material elastic coefficient.

Combined with the DAS strain–phase response model, a fully coupled model of blockage disturbance, pipeline strain, optical fiber strain, and DAS phase change can be established:

(7) $$\Delta \phi =K\cdot \eta \cdot \varepsilon_{p}(t)$$

In the dynamic monitoring of blockage evolution, the DAS phase change rate exhibits higher sensitivity to transient flow-induced disturbance, and the relationship between the phase change rate and the strain change rate is:

(8) $$\frac{d\phi }{dt}=K\cdot \eta \cdot \frac{d\varepsilon_{p}(t)}{dt}$$

To compensate for the inherent limitations of standalone DAS technology in fixed-point quantitative accuracy and high-frequency transient response, this study develops a multi-sensor collaborative perception system integrating DAS, FBG sensors, and accelerometers. All three sensors respond to the same blockage-induced vibration excitation and exhibit inherent theoretical similarities in their sensing mechanisms.

FBG sensors measure structural strain via Bragg wavelength modulation. According to fiber grating sensing theory, axial strain applied to the grating induces a drift in its central wavelength, and the strain–wavelength response relationship is expressed as [33]:

(9) $$\frac{\Delta \lambda_{B}}{\lambda_{B}}=(1-P_{e})\cdot \varepsilon_{f}(t)$$

where $\lambda_{B}$ is the central wavelength of FBG; $\Delta \lambda_{B}$ is the wavelength drift; $P_{e}$ is the effective photoelastic coefficient.

The accelerometer directly measures pipeline vibration acceleration via the piezoelastic effect. The working principle of the piezoelectric accelerometer is the following: when the accelerometer is vibrated, the force exerted by the proof mass on the piezoelectric element changes. When the measured vibration frequency is much lower than the natural frequency of the accelerometer, the change in force is proportional to the measured acceleration [34]. The relationship between acceleration and vibration displacement is:

(10) $$a(t)=\frac{d^{2}u(t)}{dt^{2}}$$

Combining Equations (4)–(10), the unified theoretical correlation formula of the three types of sensors can be obtained:

(11) $$a(t)=\frac{2}{D_{i}}\cdot \frac{d\varepsilon_{p}(t)}{dt}=\frac{2}{K\eta D_{i}}\cdot \frac{d\phi }{dt}$$

Equation (11) reveals the quantitative relationship between the pipeline vibration acceleration a(t), the pipeline surface strain rate $d\varepsilon_{p}/dt$, and the DAS phase change rate dϕ/dt. This theoretical correlation shows that DAS, FBG, and accelerometers respond to the same pipeline blockage signal source through distributed phase perception, fixed-point strain calibration, and high-frequency vibration capture, respectively. The three sensors are complementary in principle and are homologous in signal, providing theoretical support for multi-sensor, feature-level fusion and cross-validation of blockage signals.

## 3. Design of Slurry Transportation Pipeline Test System

### 3.1. Test and Monitoring System

The test platform comprised a slurry circulation system (2ZBY120/17-18.5 mining hydraulic grouting pump and JDW500 mining slurry mixer, Zhenjiang Great Wall Grouting Equipment Co., Ltd., Zhenjiang, China) and a 130.71 m pipeline, consisting of 89.71 m DN50 steel pipes and 40 m flexible hoses. The steel pipeline section was assembled via clamps, including 28 3 m straight pipes, three elbows (1 m outer diameter), two 0.5 m connecting pipes, and seven knife gate valves for gradient blockage simulation. The test was conducted using coal gangue slurry prepared from on-site mine gangue powder, with the physical and rheological parameters of the slurry strictly measured and controlled throughout the test to maintain stable flow conditions. The core parameters of the test slurry were as follows: mass concentration 60%, density 1700 kg/m^3^, dynamic viscosity 100 mPa·s, and particle size distribution with d_10_ = 30 μm, d_50_ = 120 μm, d_90_ = 350 μm, and a maximum particle size < 500 μm.

**Figure 2:**
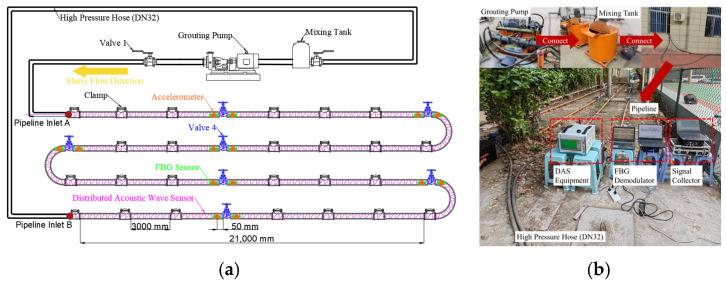
Test platform and multi-sensor monitoring system: (**a**) schematic of the coal gangue slurry circulation system; (**b**) schematic of the integrated test and monitoring system.

The monitoring system consisted of three core components: a DAS demodulation system (Wuhan Optical Valley Interconnection Technology Co., Ltd., Wuhan, China), a fiber Bragg grating (FBG) demodulation system, and a piezoelectric acceleration data acquisition unit. For the DAS system, SMG.625b single-mode optical fiber (Beijing Xizhuo Information Technology Co., Ltd., Beijing, China) was used as the sensing medium, which was helically wound at 45° along the outer wall of the steel pipeline with a total length of 201.00 m. The key technical parameters of the DAS system were as follows: a spatial resolution of 1 m, a maximum sensing distance of 50 km, a phase detection sensitivity of 1 nε/√Hz, and a sampling frequency of 1 kHz. A 1 m length of redundant fiber was reserved at each valve position to clearly distinguish vibration signals from the regions before and after the blockage point and to minimize cross-interference between adjacent sensing channels. FBG sensors (strain sensitivity 1 με, wavelength accuracy 1 pm, frequency range 0–2 kHz) were mounted at the same positions as the accelerometers. YMC121A100 piezoelectric accelerometers (Yangzhou YMC Measurement Technology Co., Ltd., Yangzhou, China) (sensitivity 10 mV/(m/s^2^), frequency range 0.5 Hz–5 kHz) were installed 50 mm upstream and downstream of each valve on the pipe outer wall, matched with a YMC9800 dynamic data acquisition unit (Yangzhou YMC Measurement Technology Co., Ltd., Yangzhou, China) for synchronous signal collection. Schematic diagrams and photographs of the test platform and monitoring system are shown in Figure 2.

To guarantee strict time synchronization, a universal trigger module output a unified 1 kHz synchronous signal for all sensors, achieving a synchronization error within 0.5 ms. The DAS channels matching the installation positions of point-type sensors were pre-calibrated via the tapping test, establishing accurate spatial mapping between distributed and discrete measurements.

To compensate for the inherent limitations of standalone DAS technology in fixed-point quantitative accuracy and high-frequency transient response, a collaborative multi-sensor system integrating DAS, FBG sensors, and accelerometers was constructed. The three core complementary principles are elaborated as follows:

(1) Spatial complementarity: DAS enables full-pipeline distributed monitoring with 1 m spatial resolution, while FBG sensors and accelerometers provide high-precision discrete measurements at key vulnerable positions, resolving the contradiction between long monitoring distance and local measurement precision.
(2) Mechanism complementarity: DAS detects strain-induced phase variations, FBG directly acquires high-precision axial strain, and accelerometers capture vibration acceleration. All signals derive from the identical blockage-induced vibration excitation, theoretically validated by Equation (11), enabling reliable cross-sensor data verification.
(3) Frequency complementarity: DAS exhibits superior sensitivity to low-frequency resonant vibrations (0.1–100 Hz), whereas accelerometers efficiently capture high-frequency transient impact signals (0.5 Hz–5 kHz). This design achieves full-band acquisition of blockage-induced vibrations, covering steady resonance and transient flow impacts.

In this collaborative system, DAS undertakes global distributed perception for pipeline vibration monitoring and blockage localization; FBG acts as a fixed-point calibration benchmark to correct DAS quantitative deviations via high-precision strain data; accelerometers supplement high-frequency transient information to remedy DAS’s insufficient high-frequency sensitivity.

Correspondingly, the subsequent data processing focuses on two core tasks: (1) extracting blockage-sensitive characteristic frequencies via multi-domain analysis and cross-verifying features with multi-sensor datasets; (2) constructing a quantitative inversion model linking DAS phase change rate and blockage degree, calibrating model parameters with FBG strain and accelerometer amplitude to realize quantitative evaluation of blockage severity.

### 3.2. Pipeline Blockage Simulation

**Figure 3:**
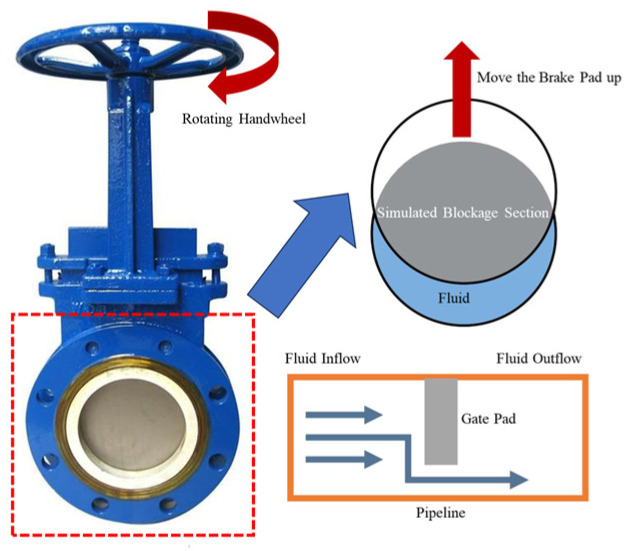
The schematic diagram of the simulated blockage of the knife gate valve.

Different blockage degrees were simulated by controlling the opening of the knife gate valve to achieve local throttling; the valve structure is shown in Figure 3.

The valve’s maximum opening was set to 10 rotations in the experiment. By rotating the handwheel 2, 4, 6, 8, and 10 circles sequentially, the blockage degree was calculated as the ratio of the effective flow area after valve throttling to the pipe cross-sectional area, corresponding to 8.76%, 23.58%, 40.94%, 59.06%, and 76.42%, respectively. Combined with the unblocked condition (0%), a total of six groups of gradient blockage test conditions were established.

While knife gate valve throttling enables precise, repeatable simulation of blockage (0–76.42%) and is essential for isolating fundamental fluid–structure interaction mechanisms, it does not fully replicate the complex, irregular, and time-evolving nature of real sedimentation blockages. Actual field blockages are asymmetric, rough-surfaced, and dynamically evolving, potentially introducing additional frequency components. However, both share the core physical consequence: a localized reduction in the flow area, which induces flow-induced vibration. Thus, the fundamental mechanisms and characteristic frequencies revealed are expected to be transferable. This method is widely adopted in pipeline blockage research [35].

### 3.3. System Localization

**Figure 4:**
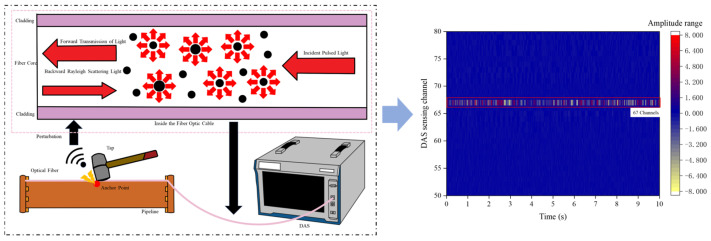
DAS tapping positioning results.

The demodulated signals from the MS-DAS system are displayed in a time–channel–phase–change–rate format. To precisely determine the blockage location, a tapping method was employed to identify the DAS channel corresponding to each valve position prior to experimentation. According to the DAS principle, tapping a specific location on the optical fiber causes an abrupt change in its refractive index, which appears on the DAS system interface as a marked increase in the signal amplitude for the relevant channel. Position verification was conducted using Valve 4 as a representative example, as shown in Figure 4. The DAS localization channel results for each valve and key pipeline position are provided in Table 1. The DAS system offers a spatial resolution of 1 m, with a positioning error of less than ±1 channel.

### 3.4. Measurement of Natural Frequency of the Grouting Delivery System

**Figure 5:**
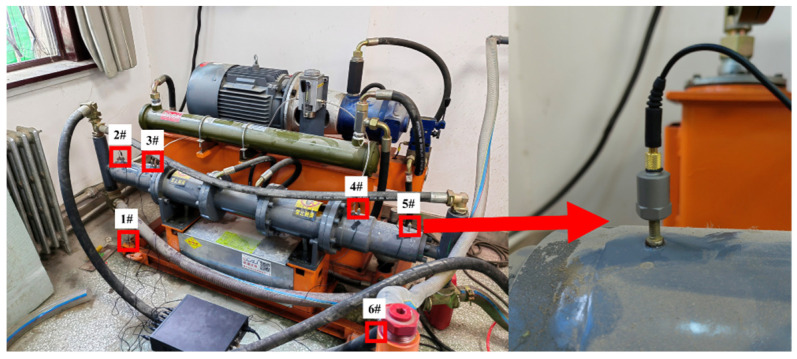
Grouting pump schematic layout of surface-mounted accelerometers.

The circulating slurry within the pipeline is propelled by a grouting pump, whose natural vibration frequency constitutes the primary source of background vibration in the system. To enable effective filtering and analysis of DAS signals, the natural vibration frequency of the grouting pump was measured using accelerometers. Six YMC121A100 unidirectional accelerometers were securely mounted with screws at key locations, including the grouting pump housing and base, as illustrated in Figure 5. Vibration signals were collected using a YMC92 (Yangzhou YMC Measurement Technology Co., Ltd., Yangzhou, China) dynamic data acquisition unit, with a sampling frequency of 1 kHz and a sampling duration of 3 min.

**Figure 6:**
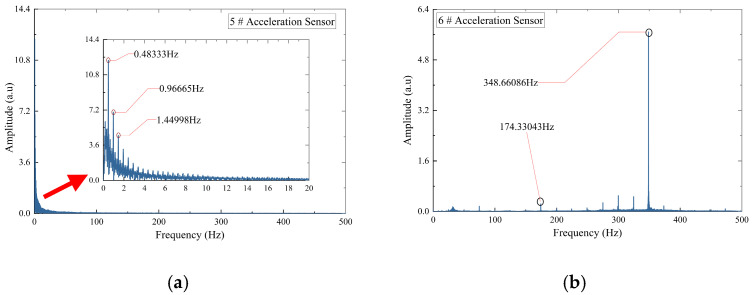
Fourier spectrum of acceleration without blockage: (**a**) Accelerometer No. A5; (**b**) Accelerometer No. A6. The amplitude is in arbitrary units (a.u.), representing the relative intensity of the vibration signal.

In the absence of blockage, Figure 6 displays the Fourier spectra of accelerometers No. 5 and No. 6. Low-frequency energy peaks are detected at 0.48 Hz, 0.97 Hz, and 1.45 Hz. In the high-frequency range, a prominent peak occurs near 348 Hz, accompanied by a secondary peak near 174 Hz. Since 348 Hz is precisely twice the 174 Hz component, this pattern suggests a typical harmonic phenomenon in mechanical structures [36]. Consequently, the primary natural vibration frequency of the grouting pump base is determined to be 174 Hz, with its second harmonic at 348 Hz.

**Figure 7:**
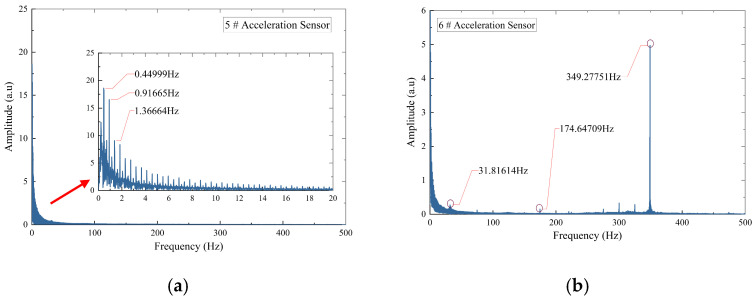
Fourier spectrum of acceleration when Valve 4 is blocked by 40.94%: (**a**) Accelerometer No. A5; (**b**) Accelerometer No. A6.

Figure 7 presents the Fourier spectrum of the acceleration signal under the 40.94% blockage condition. Low-frequency signal peaks are observed within the 0–1.5 Hz range, while high-frequency peaks persist at 174 Hz and 348 Hz, with an additional component at 31.82 Hz. These results demonstrate that the pipeline blockage does not affect the inherent natural vibration frequencies of the grouting pump.

A 4th-order Butterworth band-stop filter was implemented during signal preprocessing to remove interference from the inherent steady-state vibration of the grouting pump in subsequent data analysis. This filter was applied to both DAS and accelerometer signals, with stopband frequencies set at 0.48 Hz, 31 Hz, 49 Hz, 74 Hz, and 174 Hz. Each target frequency was assigned a narrow stopband bandwidth of ±0.2 Hz.

## 4. Experimental Results and Multi-Sensor Feature Analysis

Drawing upon the theoretical framework outlined in Section 2, a standardized multi-domain analysis was performed. All sensors were sampled at 1 kHz, corresponding to a Nyquist frequency of 500 Hz. Analog signals underwent low-pass filtering using an eighth-order Butterworth filter with a 400 Hz cutoff prior to digitization to prevent aliasing. Data processing and analysis were performed using MATLAB (R2023b, The MathWorks Inc., Natick, MA, USA) and OriginPro (2026 (64-bit) 10.3.0.180, learning edition, OriginLab Corporation, Northampton, MA, USA).

Two preprocessing workflows were designed for different analytical purposes:

Workflow 1 (for frequency-domain analysis using FFT): Preprocessing included the band-stop filter described in Section 3.4, removal of direct current (DC) components, and detrending. This workflow was used for extracting steady-state frequency components from the signals.

Workflow 2 (for time–frequency analysis using CWT): Preprocessing included only 400 Hz low-pass filtering (to prevent aliasing), DC component removal, and detrending, without the band-stop filter. CWT is designed to capture non-stationary transient signals; applying narrow band-stop filtering could potentially attenuate or distort such signals, so it was intentionally omitted from this workflow to preserve the complete information of any transient events that may exist across all frequencies.

The subsequent analysis comprised the following methods: (1) time-domain analysis, which assessed peak-to-peak and average amplitudes of phase change rate, strain, and acceleration; (2) frequency-domain analysis, utilizing fast Fourier transform (FFT) with a Hanning window (1024 points, 50% overlap, approximately 1 Hz resolution); and (3) time–frequency analysis, employing continuous wavelet transform (CWT) with a Morlet wavelet (scale 1 to 512, covering 0.5 to 500 Hz) to capture transient impacts.

### 4.1. DAS Signal Characteristic Analysis Under Normal Pipeline Transportation

#### 4.1.1. Time-Domain Characteristic Analysis

After normal system startup, the average flow rate at the pipeline inlet was 1.5 m^3^/h, with an average flow velocity of 0.2 m/s. The inlet pressure remained stable at 0.1 MPa. Channels 65 to 72, located near Valve 4, were selected for analysis of the original phase signals during both system shutdown and startup. The analysis examines the time-domain and frequency-domain characteristics of DAS signals under standard transportation conditions.

**Figure 8:**
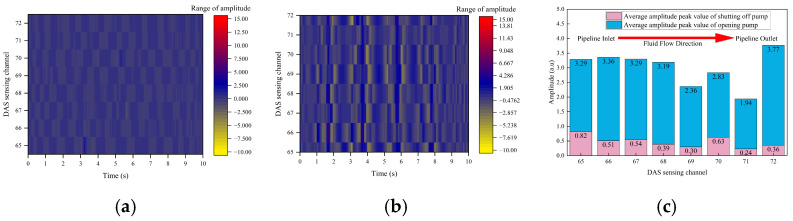
Time-domain results of the DAS signal under the on–off pump state of the pipeline system: (**a**) system pump-off state; (**b**) system pump-on state; (**c**) comparison of peak averages.

Figure 8 presents the time-domain results for DAS signals in channels 65–72 near Valve 4 during pump system shutdown and startup. In the shutdown state, the spatiotemporal distribution exhibits a uniform, low-amplitude background that serves as the baseline for system noise. Sporadic, weak-amplitude fluctuations result from random pipeline vibrations or thermal noise in the optical fiber and lack temporal regularity. This baseline enables subsequent evaluation of the signal-to-noise ratio (SNR) and identification of vibration periods during pump startup, as shown in Figure 8a.

During pump startup, the emergence of light and dark stripes in the signal represents coherent phase changes induced by vibration, reflecting the transmission and temporal evolution characteristics of pipeline vibration. In the initial startup stage (0–2 s), phase fluctuations are disordered and lack a fixed pattern, indicating the system’s transition from a transient to a steady state. In the stable operation stage (2–10 s), pronounced periodic phase fluctuations are observed, with each light–dark cycle lasting approximately 1 s. This duration provides an initial estimate of the grouting pump’s operating cycle, as shown in Figure 8b. Amplitude differences between channels indicate spatial attenuation of vibration along the pipeline, while all channels exhibit consistent fluctuation periods without significant drift during the stable stage.

Figure 8c presents the average phase-peak values for each channel under both pump shutdown and startup conditions. During startup, the average amplitude peak values are distributed smoothly across channels, with minimal temporal fluctuations, confirming the stability of the steady vibration period. Calculating the amplitude difference between startup and shutdown conditions identifies the channels most sensitive to pump vibration, which informs the optimization of key blockage-monitoring points. Variations in background noise among channels during shutdown indicate inconsistent sensitivity across sensing channels. The amplitude of channel 72 increases abnormally during startup, likely due to enhanced local eddy currents, secondary flow, and pressure pulsation resulting from abrupt changes in fluid flow direction at the elbow. Notably, this channel continues to exhibit a vibration period synchronized with the pump body, indicating that local fluid disturbances modulate vibration intensity without affecting the primary vibration period.

Comprehensive analysis of time-domain characteristics during pump shutdown and startup conditions enables effective differentiation between shutdown noise and startup vibration signals.

#### 4.1.2. Frequency-Domain Characteristic Analysis

**Figure 9:**
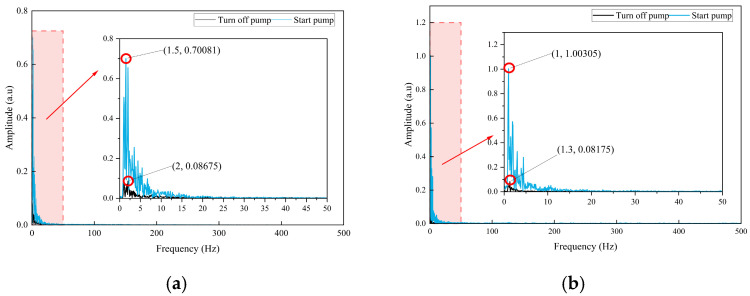
DAS phase change rate spectrum before and after the blocking point: (**a**) in front of the blockage point; (**b**) behind the blockage point.

Spectral analysis was performed on DAS channel signals before and after the Valve 4 blockage point under unblocked conditions to quantitatively characterize the system’s normal operating state. Figure 9 presents a comparison between pump-off and pump-only conditions. In the pump-off state, the signal displays background noise with dispersed spectral energy. The peak frequencies before and after the blockage point are 2 Hz and 1.3 Hz, respectively, with corresponding amplitudes of 0.08675 rad/s and 0.008175 rad/s, which are attributed to environmental vibration and intrinsic system noise.

After pump startup, spectral energy is concentrated in the low-frequency range, with a stable peak at 1.5 Hz. The amplitudes before and after the blockage point are 0.70081 rad/s and 1.00305 rad/s, representing increases of 8.08 times and 122.7 times, respectively, compared to the pump-off state. The slight frequency shift to 1 Hz downstream of the blockage point is attributed to energy attenuation during vibration transmission along the pipeline. The clear distinction between disordered background noise and a stable 1.5 Hz characteristic signal provides a reliable frequency-domain basis for objectively and accurately distinguishing between the system’s startup and shutdown states.

### 4.2. Evolution Law of DAS Signals Under Different Blockage Degrees

Valve 4 was selected as the research subject, and six incremental blockage conditions (0%, 8.76%, 23.58%, 40.94%, 59.06%, and 76.42%) were simulated. The evolution of DAS phase change rate signals was examined across the time, frequency, and time–frequency domains. Furthermore, the relationship between DAS signals and the extent of blockage was systematically investigated.

#### 4.2.1. Nonlinear Evolution of Time-Domain Amplitude

**Figure 10:**
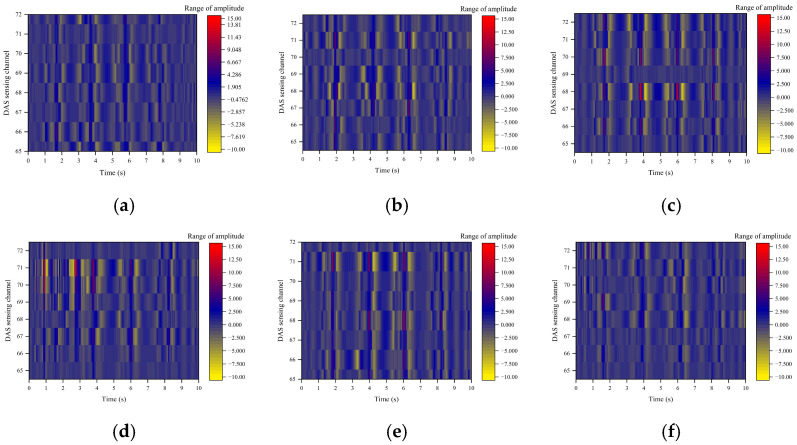
Time-domain waterfall diagram of the DAS phase change rate: (**a**) no blockage; (**b**) blockage degree 8.76%; (**c**) blockage degree 23.58%; (**d**) blockage degree 40.94%; (**e**) blockage degree 59.06%; (**f**) blockage degree 76.42%.

Figure 10 presents time-domain waterfall diagrams of the DAS phase change rate in channels 65 to 72 near Valve 4 under varying blockage degrees. The horizontal axis indicates time (s), the vertical axis denotes DAS channels, and the color scale represents the amplitude of phase change rate (unit: a.u.).

Based on the analysis of the spatiotemporal distribution characteristics of the DAS phase change rate, the blockage evolution process can be divided into four distinct stages:

(1) Unblocked reference state (0%): The system displays a uniform dark blue background, and the phase change rate in each channel remains at the background noise level. Pipeline flow is stable, exhibiting minimal turbulence and pressure pulsation. This state serves as a benchmark for identifying and comparing subsequent blockage conditions.
(2) Mild blockage stage (8.76–23.58%): Faint light-colored fluctuations are observed in individual channels adjacent to the blockage location. The signal amplitude remains low, spatial influence is limited, and the flow disturbance is weak. The signal-to-noise ratio (SNR) of DAS signals is also low, which complicates effective identification.
(3) Moderate blockage stage (40.94%): The amplitude and spatial diffusion range of phase change rate fluctuations reach their maximum. High-amplitude responses (dark red/red) are evident in channels corresponding to the blockage, with fluctuations extending to multiple consecutive downstream channels. At this stage, the pipeline flow area continues to support relatively high flow velocity. The choking effect at the blockage induces intense turbulence and pressure pulsations, thereby maximizing the optical fiber’s strain response.
(4) Severe blockage stage (59.06–76.42%): The fluctuation amplitude and spatial range decrease significantly compared to the peak stage, approaching the low-disturbance state observed during mild blockage. The severely restricted flow area results in a sharp decline in slurry flow rate, causing fluid stagnation and a corresponding reduction in flow disturbance intensity.

The time-domain waterfall diagram provides an intuitive representation of DAS signal characteristics during pipeline blockage. However, it only qualitatively indicates fluctuation trends, does not accurately quantify disturbance intensity across different stages, and makes precise localization of the blocking point challenging.

To quantify the nonlinear evolution of the DAS phase change rate with blockage degree, the average peak amplitude from channels 65–72 was calculated (covering the front and rear of the Valve 4 blockage point, corresponding to the localization results in Table 1), as shown in Table 2. The average peak amplitude increases from 1.905 a.u. at 8.76% blockage to 13.81 a.u. at 40.94% blockage and then decreases to 2.857 a.u. at 76.42% blockage, which quantitatively verifies the unimodal trend of the DAS time-domain response. The maximum amplitude at 40.94% blockage is 7.25 times that at 8.76% blockage and 4.84 times that at 76.42% blockage, revealing that the blockage-induced flow disturbance reaches its peak at 40.94% blockage in the time domain.

The time-domain waterfall diagram enables intuitive visualization of the spatiotemporal evolution of blockage-induced vibration, while the quantitative analysis in Table 2 provides a data foundation for subsequent blockage feature extraction. However, time-domain analysis alone cannot distinguish the physical sources of vibration signals, nor can it achieve precise blockage localization.

#### 4.2.2. Extraction and Response of Frequency-Domain Characteristic Frequencies

**Figure 11:**
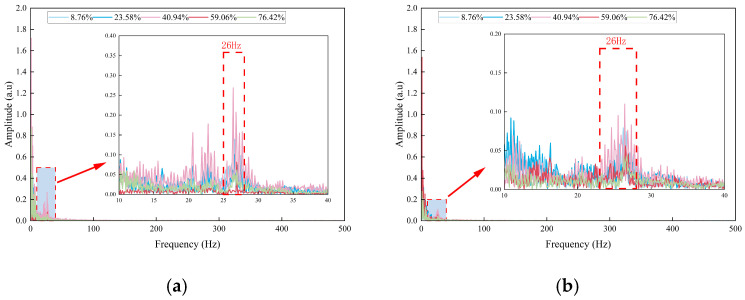
Spectrum of DAS phase change rate before and after the blockage point with different blockage degrees: (**a**) in front of the blockage point; (**b**) behind the blockage point.

A comparison of the frequency spectra under the conditions of “only pump on without blockage” and “different degrees of blockage with pump on” indicates that, in the blocked state, a stable characteristic frequency peak at 26 Hz emerges in addition to the 1.5 Hz vibration. This peak is observed exclusively during blockage, as illustrated in Figure 11.

The 26 Hz characteristic frequency is directly linked to fluid–structure interaction resonance caused by blockage. As fluid passes through the blockage, the sudden reduction in effective flow area leads to a significant increase in local flow velocity and shear-layer instability. This process initiates blockage-induced high-frequency turbulent pulsations and vortex shedding. From a system dynamics perspective, pipeline blockages introduce an additional local mass, substantially altering the structure’s dynamic properties [37].

To further confirm that the 26 Hz component arises from fluid–structure interaction resonance, a simplified theoretical estimation of the natural frequency of the local valve–pipe assembly was conducted. The pipe segment adjacent to the valve was modeled as a clamped–clamped Euler–Bernoulli beam with an effective length of 1.2 m, an outer diameter of 60 mm, and a wall thickness of 5 mm. Using the material properties of steel (*E* = 206 GPa, ρ = 7850 kg/m^3^), the fundamental transverse natural frequency was calculated to be approximately 25.7 Hz, which closely aligns with the observed 26 Hz peak. This result strongly suggests that the 26 Hz frequency corresponds to a structural natural frequency of the local assembly.

Simultaneously, the vortex shedding frequency induced by the blockage was estimated using the Strouhal relation $f_{v}=S_{t}\cdot U/D$ [38]. For the main pipe flow (U = 0.2 m/s, *D* = 50 mm, $S_{t}$ ≈ 0.2), the calculated vortex shedding frequency in the mainstream is only 0.8 Hz. Even under extreme throttling conditions with a 76.42% blockage degree, the maximum vortex-shedding frequency at the throttle port is only 4.8 Hz, which is still an order of magnitude lower than 26 Hz. These findings definitively rule out the possibility that the 26 Hz frequency results solely from flow-induced vortex shedding.

In this experiment, the vortex shedding frequency approaches the natural frequency of the local valve–pipeline structure, which initiates fluid–structure interaction resonance. This resonance manifests as a stable and prominent 26 Hz peak in the frequency domain. Therefore, the presence of the 26 Hz characteristic frequency can serve as a deterministic indicator of pipeline blockage in the frequency domain, providing a critical feature for early detection.

**Figure 12:**
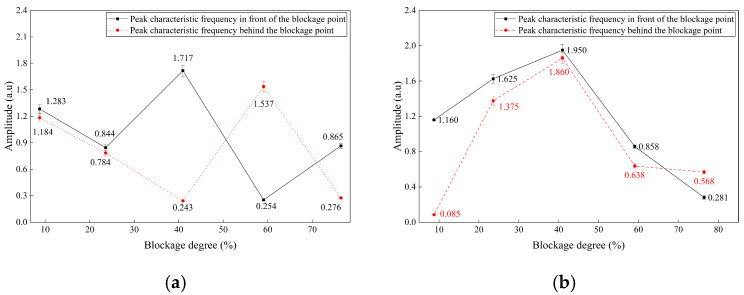
Variation in the peak value of the characteristic frequency before and after the blocking point: (**a**) amplitude at 1.5 Hz characteristic frequency; (**b**) amplitude at 26 Hz characteristic frequency.

To quantify the intensity of blockage-induced disturbances and their spatial transmission characteristics, the variations in amplitudes of the 1.5 Hz and 26 Hz characteristic frequencies relative to the degree of blockage were analyzed, as shown in Figure 12.

The amplitude of the 1.5 Hz pump-dominated frequency initially decreases, subsequently increases, and then decreases again. During the mild blockage stage (8.76% to 23.58%), a slight amplitude reduction occurs due to increased fluid resistance and associated damping. At the moderate blockage stage (40.94%), the amplitude reaches its maximum, corresponding to the strongest fluid–structure coupling. In the severe blockage stage (59.06% to 76.42%), the amplitude declines rapidly as the coupling effect diminishes with reduced slurry flow rate under high blockage conditions. The amplitude measured ahead of the blockage point consistently exceeds that measured behind it, demonstrating spatial attenuation of vibration energy along the pipeline.

The amplitude of the 26 Hz characteristic frequency exhibits a similar unimodal trend, peaking at 40.94% blockage. This trend directly reflects variations in the intensity of turbulent pulsations induced by blockage. Pulsation intensity increases gradually during the mild blockage stage, attains a maximum due to pronounced fluid–structure interaction in the moderate blockage stage, and decreases markedly as fluid stagnation occurs in the severe blockage stage. This frequency characteristic enables effective differentiation between the low-disturbance states in the mild and severe blockage stages, which otherwise present similar time-domain behavior. Specifically, the 26 Hz amplitude is moderate at 8.76% blockage and declines to a very low level at 76.42% blockage. Additionally, the spatial distribution of the 26 Hz amplitude, with higher values ahead of the blockage point and lower values behind it, serves as a key frequency-domain indicator for locating the blockage point.

Measurement repeatability and reliability were assessed by conducting each blockage test three times under identical operating conditions. The error bars in Figure 12 represent the ± standard deviation (SD) of the repeated measurements, with a maximum relative standard uncertainty of 3.6% for all characteristic frequency amplitudes. The minimal dispersion and low uncertainty demonstrate that the extracted characteristic frequency amplitude data are highly stable and reproducible.

The frequency-domain analysis results closely correspond to the nonlinear fluctuation trend of “first increasing and then decreasing” observed in the time-domain analysis. This cross-domain consistency validates the reliability of multi-domain features and provides dual indicators for the qualitative identification of pipeline blockage.

#### 4.2.3. Transient Impact Characteristics Based on Continuous Wavelet Transform

**Figure 13:**
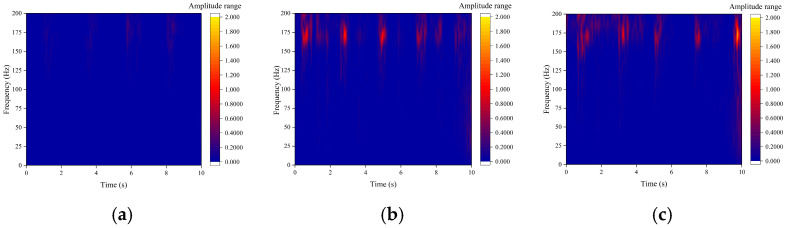

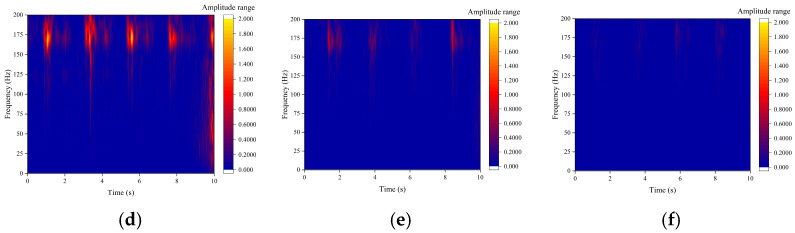
The wavelet transform results of different degrees of blockage: (**a**) no blockage; (**b**) blockage degree 8.76%; (**c**) blockage degree 23.58%; (**d**) blockage degree 40.94%; (**e**) blockage degree 59.06%; (**f**) blockage degree 76.42%.

The pipeline phase change rate signals collected by DAS are non-stationary and complex. The fast Fourier transform (FFT) provides only the cumulative energy distribution of global frequency components and does not capture the dynamic evolution of frequency over time. Therefore, the continuous wavelet transform (CWT) was employed for time–frequency domain analysis to extract transient impact characteristics induced by blockage accurately and to investigate their correlation with the degree of blockage. CWT was applied to the DAS phase change rate signals upstream of the blockage point. The results are presented in Figure 13, where the horizontal axis indicates time (s), the vertical axis indicates frequency (Hz), and the color scale represents signal energy (a.u.).

In the unblocked state (Figure 13a), the time–frequency diagram shows a uniform energy distribution with very low amplitude and no distinct characteristic frequencies. In contrast, the blocked state (Figure 13b–f) exhibits a pronounced transient high-energy distribution in the time–frequency diagram, with a central frequency consistently near 174 Hz, as indicated by intermittently bright energy bands. This pattern signifies the transient impact characteristic frequency associated with pipeline blockage. The dominant 26 Hz component observed in the FFT spectrum manifests as a continuous, low-energy distribution band in the time–frequency diagram.

To quantitatively assess the energy difference between the two frequency components, the instantaneous energies at 26 Hz and 174 Hz were extracted from the continuous wavelet transform (CWT) time–frequency diagrams. The peak energy of the 174 Hz component at specific transient moments reaches approximately 1000 a.u., which is two orders of magnitude greater than the average energy of the 26 Hz component during the entire blockage period (approximately 10 a.u.); this substantial difference in instantaneous energy accounts for the distinct patterns observed in the time–frequency diagrams.

The 174 Hz transient impact frequency corresponds to the natural vibration frequency of the grouting pump base measured in Section 3.4. Notably, two distinct signals coexist in this band: the pump’s inherent steady-state vibration (a continuous low-energy background) and the detected intermittent high-energy bursts induced by blockage. This is supported by three key observations: (1) the bursts only emerge under blocked conditions and correlate positively with blockage degree; (2) the grouting pump operated at a constant speed, flow rate, and inlet pressure throughout all tests (with no load-dependent modulation or speed variations), directly eliminating the possibility that changes in pump operation caused the energy bursts; (3) fluid collisions and cavitation at the blockage site naturally excite the structure at its inherent natural frequency.

### 4.3. Multi-Sensor Cross-Validation and Comparative Analysis

While DAS technology enables distributed monitoring, it exhibits limitations in fixed-point precision and quantitative accuracy. To verify the physical authenticity and reliability of the three core characteristic frequencies (1.5 Hz, 26 Hz, and 174 Hz) identified previously, FBG sensors and piezoelectric accelerometers were incorporated for point-type synchronous monitoring. This approach established a multi-dimensional sensing system comprising distributed strain (DAS), local fixed-point strain (FBG), and point-type vibration acceleration (accelerometer). Comparative analysis of monitoring signals from the three sensor types in the time, frequency, and time–frequency domains enabled cross-validation of the blockage characteristic frequencies. It clarified the respective monitoring advantages and limitations of each sensor.

#### 4.3.1. FBG Sensor Strain Response Validation

FBG sensors utilize wavelength modulation and offer several advantages, including high-precision fixed-point strain measurement (resolution of 1 με), immunity to electromagnetic interference, and the ability to compensate for temperature–strain cross-sensitivity [39]. These sensors can validate DAS-distributed monitoring results by quantifying local strain and detecting pipeline microstructural deformation caused by blockages, thereby establishing a complementary system for distributed positioning and fixed-point quantification. FBG sensors are positioned at DAS tapping points located before and after the blockage.

(1) Time-Domain Characteristic Analysis

**Figure 14:**
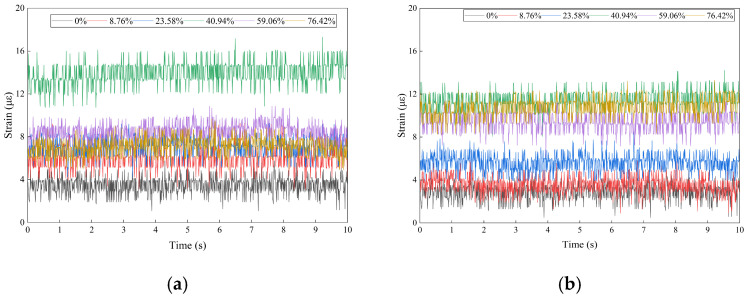
FBG strain time-domain diagram before and after different degrees of blockage: (**a**) in front of the blockage point; (**b**) behind the blockage point.

Figure 14 presents the time-domain FBG strain signals measured before and after the blockage point for varying degrees of blockage. The horizontal axis represents time (s), and the vertical axis represents axial strain (μϵ). The FBG strain signals display layered fluctuations that are primarily determined by the degree of blockage, with distinct spatial differences. The fluctuation frequencies are fully synchronized with the DAS signals, with a period of approximately 2 s, corresponding to the 1.5 Hz system fundamental frequency. Notably, FBG signals demonstrate a narrower fluctuation range, indicating strong resistance to interference. Even at a high blockage level of 76.42%, the standard deviation of FBG strain fluctuations is only 0.8 μϵ, significantly lower than 1.2 a.u. observed in DAS signals. These results confirm that FBG sensors can reliably capture local strain signals in complex vibration environments.

During normal transportation, the FBG axial strain measured before and after the blockage point remains stable at 3 to 5 με, with minimal fluctuations, indicating only minor influence from the steady-state vibration of the grouting pump. As the blockage degree increases from 8.76% to 76.42%, the strain amplitude follows a nonlinear trend similar to that observed in DAS signals, reaching a maximum at 40.94% blockage. The strain in front of the blockage increases to 6–9 με, a 60–80% rise, while the strain behind the blockage increases to 4–6 με. Under moderate blockage conditions, the strain in front peaks at 15 με (a 200% increase), and the strain behind reaches 12 με (a 140% increase). The “throat high-speed jet effect” is pronounced at this stage, and the pipeline’s structural deformation is at its maximum. In the severe blockage stage, the strain decreases to 7 to 8 με in front and 8 to 10 με behind the blockage.

The high fixed-point precision of FBG sensors enables detection of subtle local strain-gradient differences. In contrast, DAS distributed monitoring is constrained by its 1 m spatial resolution and cannot distinguish strain changes occurring at 50 mm intervals. Therefore, the two methods are complementary.

(2) Frequency-Domain Characteristic Analysis

**Figure 15:**
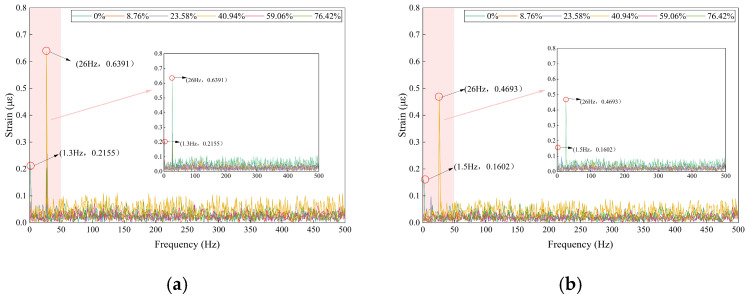
FBG strain frequency-domain diagram before and after different degrees of blockage: (**a**) in front of the blockage point; (**b**) behind the blockage point.

The fast Fourier transform (FFT) was applied to the FBG strain signals, with the results presented in Figure 15. The spectral characteristics are strongly correlated with blockage conditions and closely align with distributed acoustic sensing (DAS) signals. Both exhibit a bimodal structure consisting of a low-frequency background peak and a 26 Hz blockage characteristic peak, thereby enabling cross-validation of the 26 Hz characteristic frequency.

In the absence of blockage, only a low-frequency background peak near 1.5 Hz is observed in the frequency spectrum, with amplitudes of 0.2155 με and 0.1602 με before and after the blockage point, respectively. This observation corresponds to the 1–2 Hz low-frequency band in the DAS frequency domain and reflects the fundamental excitation effect of grouting pump vibration. Furthermore, this peak remains unaffected by the degree of blockage and serves exclusively as a background signal for the system, confirming the inherent vibration stability of the test equipment.

When the pipeline is blocked, a narrowband characteristic peak at 26 Hz emerges in the frequency spectrum. The amplitude variation in this peak closely corresponds to that observed in DAS, reaching a maximum at 40.94% blockage, with amplitudes of 0.6394 με and 0.4693 με before and after the blockage point, respectively. As the blockage degree increases to 76.42%, the 26 Hz amplitude decreases to approximately 0.25 με. As a point-type sensing technology, FBG provides higher quantitative accuracy for the 26 Hz characteristic peak and can directly detect micro-strain fluctuations at this frequency. In contrast, while DAS can accurately determine the spatial location of the 26 Hz characteristic peak, its fixed-point quantitative accuracy is somewhat lower. These technologies thus form a complementary relationship, combining spatial positioning with fixed-point quantification and further confirming that 26 Hz is a common characteristic frequency for pipeline blockage.

This comparison demonstrates the complementary roles of DAS and FBG in blockage monitoring. DAS enables distributed spatial detection of blockage regions, while FBG provides high-precision quantitative calibration of the blockage degree. Together, these capabilities establish the foundation for a multi-sensor fusion framework.

#### 4.3.2. Vibration Response Verification of Accelerometer

To further validate the conclusions regarding the vibration characteristics of pipeline blockage monitored by DAS, YMC121A100 accelerometers were employed under identical test conditions. The acceleration signals collected by Accelerometers 1 and 2, positioned before and after the blockage point, were analyzed in the time, frequency, and time–frequency domains. These results were then compared with those obtained from DAS and FBG signals.

(1) Time-Domain Analysis of Vibration Characteristics

**Figure 16:**
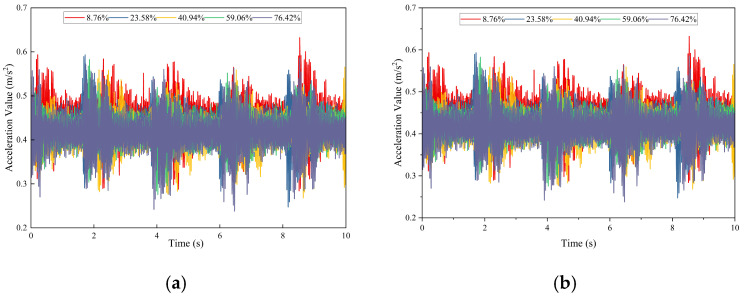
Time-domain diagram of the acceleration sensor response as a function of the degree of blockage: (**a**) Accelerometer No. 1; (**b**) Accelerometer No. 2.

Figure 16 presents the time-domain acceleration signals measured before and after the blockage point for varying degrees of blockage. The horizontal axis represents time (s), while the vertical axis denotes vibration acceleration (m/s^2^).

Across all blockage levels, the time-domain acceleration waveforms exhibit weak fluctuations with a period of approximately 2 s, which aligns with the operating cycle of the grouting pump and indicates the fundamental excitation of the equipment’s steady-state vibration. However, the curves corresponding to different blockage degrees exhibit substantial overlap; thus, blockage degree cannot be reliably distinguished from time-domain waveforms alone. Furthermore, the distinct trend of “first increasing and then decreasing” observed in FBG and DAS signals is not apparent. This limitation arises because accelerometers are insensitive to low-frequency strain disturbances caused by pipeline blockage and primarily detect the strong inherent vibration of the equipment.

(2) Frequency-Domain Characteristic Analysis

**Figure 17:**
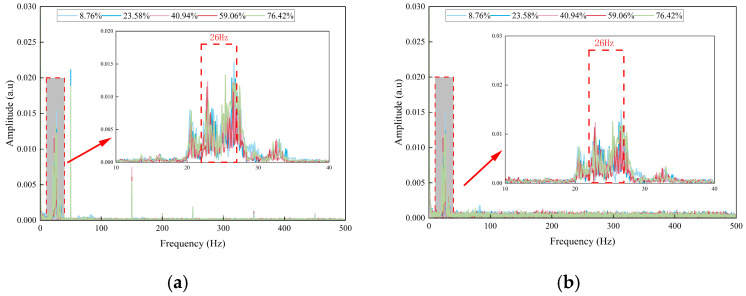
FFT results of acceleration values with different blockage degrees: (**a**) in front of the blockage point; (**b**) behind the blockage point.

The fast Fourier transform (FFT) of acceleration signals reveals a 26 Hz characteristic peak, which is consistent with DAS and FBG observations, as shown in Figure 17. This result constitutes the third cross-validation of this characteristic frequency, thereby confirming its physical authenticity.

Following pipeline blockage, a narrowband characteristic peak emerges near 26 Hz in the frequency domain of the acceleration signal. The amplitude variation in this peak aligns with those observed in the DAS and FBG signals, with the peak occurring at a blockage degree of 40.94%. Additionally, the acceleration signal’s frequency domain reveals high-frequency harmonics with a fundamental frequency of 50 Hz, a feature not prominently observed in the DAS signal. This finding suggests that the accelerometer is more sensitive to high-frequency vibrations above 50 Hz than DAS. Conversely, the amplitude of the 1–2 Hz system background fundamental frequency is extremely low in the acceleration frequency domain, indicating a much weaker low-frequency capture capability than that of DAS. These observations clearly demonstrate the differences in frequency sensitivity among the various sensors.

(3) Time–Frequency Domain Analysis

**Figure 18:**
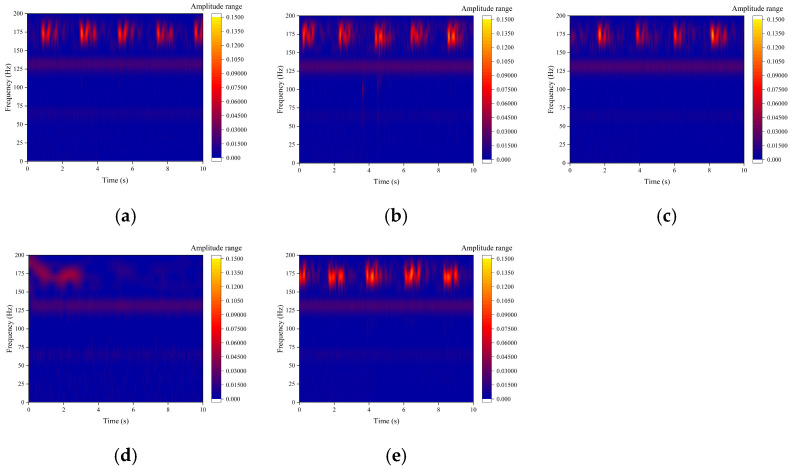
The wavelet transform in front of blockage points under different degrees of blockage: (**a**) blockage degree 8.76%; (**b**) blockage degree 23.58%; (**c**) blockage degree 40.94%; (**d**) blockage degree 59.06%; (**e**) blockage degree 76.42%.

The continuous wavelet transform (CWT) was applied to acceleration signals recorded before and after the blockage point. The results, presented in Figure 18, display time (s) on the horizontal axis, frequency (Hz) on the vertical axis, and signal energy (a.u.) as the color scale. In the blocked state, intermittent high-energy main frequency bands are observed around 174 Hz in the time–frequency diagram of the acceleration signal. These bands are fully consistent with the transient impact characteristic frequency of the DAS signal. Additionally, the temporal variation in these frequency bands closely matches that of the DAS signal, thereby confirming the cross-validation of the 174 Hz transient impact frequency.

The 174 Hz transient impact frequency band exhibits maximum energy under the 40.94% moderate blockage condition, which aligns with the observed variation in DAS signals. This finding further demonstrates that the energy variation at this frequency is directly correlated with the intensity of fluid transient disturbances caused by pipeline blockage. The grouting pump maintained strictly constant operating parameters throughout all tests, indicating that the 174 Hz energy variation is strongly associated with pipeline blockage intensity rather than pump operating status. These results confirm that the excitation source is the blockage-induced fluid transient impact rather than modulation from pump vibration.

This comparison demonstrates that the accelerometer possesses a significant advantage in capturing high-frequency transient signals compared to DAS. The accelerometer can effectively address the high-frequency monitoring limitations of DAS and enhance the detection of transient blockage risks.

#### 4.3.3. Synchronous Consistency Verification of Multiple Sensors for the 26 Hz Characteristic Frequency

Building on the previous verification results for the FBG and accelerometer, the synchronous consistency of the 26 Hz characteristic frequency extracted by DAS, FBG, and the accelerometer was quantitatively analyzed. This analysis aimed to further confirm the physical authenticity and stability of the characteristic frequency at the experimental level. The results indicate that DAS, FBG, and the accelerometer each extracted a stable 26 Hz characteristic peak synchronously under all blockage conditions (8.76–76.42%). In contrast, no such characteristic peak was observed under the unblocked condition (0%). The measured frequency values for the three sensors at different blockage degrees are presented in Table 3.

Across varying blockage levels, the measured frequency values from the three sensors are concentrated between 25.7 and 26.2 Hz. The maximum frequency deviation among the sensors did not exceed 0.4 Hz, which is significantly lower than the FFT analysis’s frequency resolution (approximately 1 Hz) used in this study. This demonstrated excellent frequency consistency. These findings confirm that the 26 Hz characteristic frequency is not attributable to random noise detected by a single sensor or to signal distortion from measurement error in any individual sensing system. Instead, it represents a genuine physical excitation signal generated by fluid–structure interaction during pipeline blockage, synchronously detected by three types of sensors employing different sensing principles.

The frequency stability of the 26 Hz characteristic peak under varying flow field conditions was verified using gradient blockage test results. As the blockage degree increased from 8.76% to 76.42%, the flow velocity, flow field structure, and pressure distribution within the pipeline changed substantially. However, the measured 26 Hz characteristic frequency consistently remained within 25.7–26.2 Hz, exhibiting no linear shift with changes in flow parameters. Only the amplitude of the characteristic peak varied with blockage degree, following the same unimodal trend described in Section 4.2, with a maximum at 40.94% blockage. This behavior aligns with the fundamental property of a structural natural frequency, rather than a flow-induced frequency that varies linearly with flow parameters. The result was entirely consistent with the theoretical calculations presented in Section 4.2.2 and provided a comprehensive theoretical and experimental demonstration of the physical mechanism underlying the 26 Hz characteristic frequency.

### 4.4. Analysis of Multi-Characteristic Frequencies and Their Implications for Blockage Signals

Time–frequency analysis and multi-sensor cross-validation identified three characteristic frequencies that are strongly correlated with pipeline blockage, establishing a hierarchical feature system for blockage monitoring. The physical mechanisms and practical implications of these frequencies are outlined below.

The 1.5 Hz fundamental frequency of the system results from the periodic operation of the grouting pump. This frequency remains stable across all blockage conditions, with only its amplitude changing, reaching a maximum at 40.94% blockage. It functions as a steady-state background reference, indicating the overall disturbance level within the pipeline.

The 26 Hz blockage resonance frequency arises from fluid–structure interaction resonance of the local valve–pipe assembly, confirmed as a structural natural frequency (theoretically 25.7 Hz via Euler–Bernoulli beam model). Its amplitude exhibits a clear unimodal trend with blockage degree, peaking at 40.94%, making it a deterministic indicator for blockage identification and quantitative severity assessment.

The 174 Hz transient impact frequency corresponds to the grouting pump base’s inherent natural frequency. During blockage evolution, transient events, such as slurry collisions and local cavitation, excite this frequency, producing intermittent high-energy bursts in the time–frequency domain. Its energy peaks at 40.94% blockage, serving as a sensitive indicator for capturing flow field mutations and enabling early warning of severe blockage.

Together, these three frequencies constitute a comprehensive monitoring system: 1.5 Hz serves as a steady-state background reference signal, 26 Hz facilitates continuous blockage identification and quantification, and 174 Hz detects transient flow variations. This three-frequency hierarchical feature system directly addresses the lack of verified, specific discriminants for slurry pipeline blockage monitoring, providing a solid feature foundation for subsequent blockage identification and quantitative assessment.

## 5. Pipeline Blockage Degree Quantitative Characterization and Joint Analysis

### 5.1. Joint Comparison of Multi-Sensor Feature Consistency

**Figure 19:**
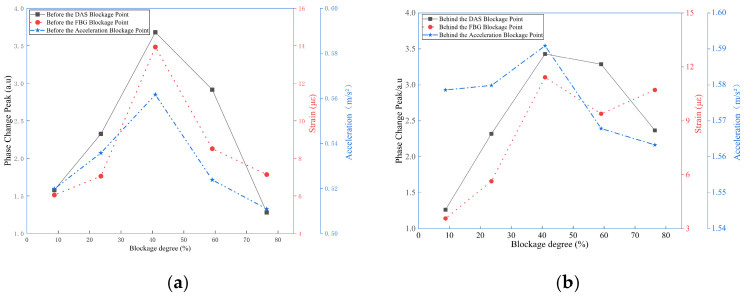
Comparison of points before and after blockage at different blockage degrees: (**a**) peak value in front of the blockage point; (**b**) peak value behind the blockage point.

The three sensors demonstrated strong consistency in detecting blockage-related features, as shown in Figure 19. The peak amplitudes of DAS phase change rate, FBG strain, and acceleration at 26 Hz all vary systematically with the degree of blockage, reaching a maximum at 40.94% blockage. This synchrony indicates that the signals arise from the same physical mechanism, specifically blockage-induced fluid–structure interaction, thereby supporting the rationale for multi-sensor fusion.

At a 40.94% moderate blockage condition, the DAS phase change rate amplitude measured in front of the blockage point is 1.7168 a.u., the FBG strain peak is 15 με, and the acceleration amplitude is 0.01227 m/s^2^. All sensors record higher amplitudes in front of the blockage point than behind it, which aligns with the spatial attenuation of vibration energy along the pipeline and supports effective blockage localization.

### 5.2. Quantitative Characterization Model of Pipeline Blockage Degree Based on DAS Phase Change Rate

The primary advantage of DAS technology is its ability to provide distributed, long-distance monitoring, which meets the engineering requirements for long-distance coal gangue slurry pipelines. Consequently, a quantitative inversion model for pipeline blockage degree was developed using DAS phase change rate signals to enable distributed quantitative assessment of blockage degree.

**Figure 20:**
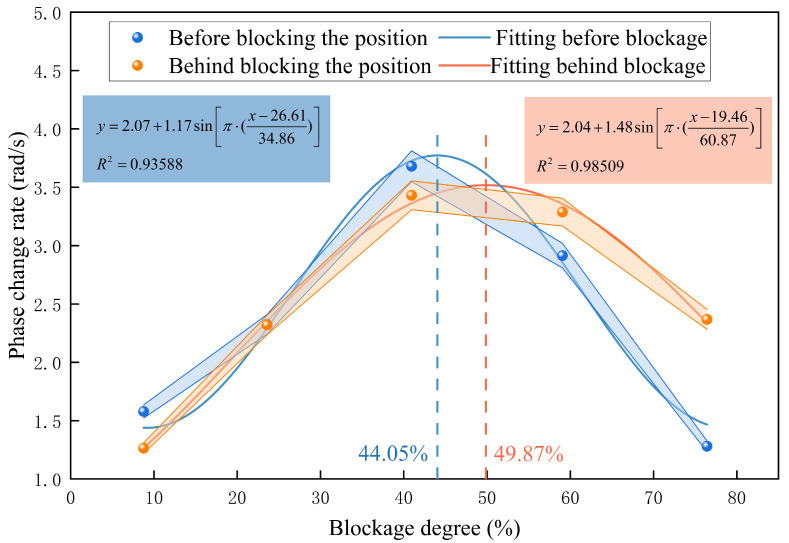
The change in phase change rate before and after the blockage point under different blockage degrees.

A statistical analysis of peak averages was conducted on the preprocessed DAS phase change rate signals to investigate the quantitative relationship between blockage degree and signal amplitude, as illustrated in Figure 20. For blockage degrees ≤ 40.94%, the average peak value of the phase change rate increases as the blockage degree increases, and the response amplitude before the blockage point consistently exceeds that after the blockage point. When the blockage degree exceeds 40.94%, the average peak value of the phase change rate decreases, and the spatial amplitude relationship reverses, with the response amplitude after the blockage point surpassing that before the blockage point.

Nonlinear fitting using a sine function was conducted separately for the relationships before and after the blockage point. The sine function was chosen for its physical consistency and superior model performance: the DAS phase change rate exhibits a smooth, unimodal trend with blockage degree, reflecting the physical progression of the blockage-induced flow disturbance and fluid–structure interaction. Alternative nonlinear models, including second- and third-order polynomials and Gaussian functions, were evaluated; the sine function provided the highest goodness of fit while maintaining simplicity with only three parameters. With six gradient blockage degrees spanning the entire test range, the model remains low in complexity and minimizes the risk of overfitting.

Least squares fitting produced quantitative inversion models for both pre- and post-blockage point relationships. Each blockage test was conducted in triplicate; error bars in Figure 20 indicate ±SD, with a maximum relative standard uncertainty of 3.6%, demonstrating high measurement stability and reproducibility. The goodness-of-fit R^2^ values are 0.936 before the blockage point and 0.985 after, with an overall maximum of 0.985. These results indicate that the sine-fitting model accurately characterizes the quantitative relationship between the blockage degree and the amplitude of the DAS phase change rate.

To quantify the relative uncertainty of the fitting model parameters, the relative standard errors (RSEs) were calculated as the ratio of the standard error to the parameter estimate. For the pre-blockage model, the RSEs are 6.3% (*y*_0_), 10.1% (*x_c_*), 13.3% (*w*), and 12.8% (A). For the post-blockage model, the RSEs are 20.5% (*y*_0_), 33.0% (*x_c_*), 20.0% (*w*), and 25.4% (A). The lower RSEs of the pre-blockage model indicate more stable parameter estimation, while the higher RSEs of the post-blockage model are attributed to the small sample size (*n* = 5) and the inherent data variability at high blockage degrees.

Model robustness was further evaluated using leave-one-out cross-validation (LOOCV). The sine model was iteratively fitted with five of the six blockage conditions and used to predict the DAS amplitude for the remaining condition, repeating this process for each blockage degree. For the post-blockage model (full-sample R^2^ = 0.985), the average cross-validation R^2^ was 0.979, only 0.006 lower than the full-sample R^2^, with a mean relative prediction error of 3.77% (<5%). These findings confirm that the model demonstrates strong generalization capability within the tested experimental range and is not significantly overfitted.

The peaks of the fitting curve corresponded to blockage degrees of 44.05% (before blockage) and 49.87% (after blockage), which were highly consistent with the experimental vibration response peak at 40.94%, thereby verifying the model’s accuracy. This sine-fitting quantitative inversion model facilitates the transition from qualitative discrimination to quantitative assessment of pipeline blockage degree, identifying the 40–50% range as the most sensitive interval for vibration response to pipeline blockage.

Nevertheless, the small number of experimental points (six gradient blockage degrees) imposes limitations on the model’s generalization ability beyond the tested blockage range. Future work will supplement additional intermediate blockage gradients to improve parameter stability and model applicability.

### 5.3. Resonance Window Mechanism and Model Applicable Boundary

The 40.94% peak blockage degree of the DAS signal response is a key finding of this study, representing a fluid–structure interaction resonance window under fixed laboratory conditions (flow velocity *U* = 0.2 m/s, pipe diameter *D* = 50 mm, slurry concentration 60%). This section focuses on its physical mechanism, the regulatory trends under variable operating parameters, and the applicability of the sine-fitting model to address concerns about the model’s universality.

#### 5.3.1. Physical Mechanism of the Resonance Window

The formation of the resonance window depends on two interdependent core conditions:

(1) Frequency matching: The turbulent pulsation and vortex shedding frequency induced by pipeline blockage matches the natural frequency (26 Hz) of the local valve–pipe assembly, which is consistent with the theoretical calculation result (25.7 Hz) of the clamped–clamped Euler–Bernoulli beam model [37].
(2) No slurry stagnation: The blockage degree remains below the level at which significant flow deceleration occurs, ensuring efficient energy transfer of fluid–structure interaction.

The unimodal nonlinear trend of the DAS response (first increasing and then decreasing with blockage degree) is an intrinsic feature of slurry pipeline blockage, independent of operating parameters, while the specific position of the resonance window is dependent on flow velocity, pipe diameter, and slurry properties. This unimodal trend results from the underlying fluid–structure interaction mechanism: increased turbulent pulsation during early blockage and reduced disturbance during severe blockage due to slurry stagnation.

Due to experimental constraints, this study is limited to the fixed operating parameters described above and does not include targeted tests under variable conditions. However, for flow velocity, pipe diameter, and slurry properties, the following regulatory tendencies can be inferred based on fluid–structure interaction theory and previous research findings [16,19,21,37]:

(1) Flow velocity: Lower flow velocity requires a higher blockage degree to trigger resonance, while higher flow velocity enables resonance at a lower blockage degree.
(2) Pipe diameter: Changes in pipe diameter alter the structural natural frequency and flow field characteristics, leading to a resonance window offset.
(3) Slurry properties: Coarse particles/low concentration may shift the window to a lower blockage degree. In comparison, fine particles/high concentration/high viscosity may shift it to a higher blockage degree, accompanied by changes in the sine-fitting model’s amplitude parameter.

#### 5.3.2. Applicable Boundary of the Sine-Fitting Model

The sine-fitting model (R^2^ = 0.985) is applicable only within the fixed experimental parameter range investigated in this study. For engineering applications outside this range, targeted calibration experiments under actual operating conditions are required to update the resonance window position and model parameters (amplitude and phase), thereby ensuring quantitative accuracy.

### 5.4. Analysis of Sensor Performance Differences and Collaborative Monitoring Framework

#### 5.4.1. Analysis of Sensor Performance Differences and Complementarity

Significant differences exist in the monitoring principles and technical parameters of DAS, FBG, and accelerometer sensors. These differences result in distinct advantages and limitations in terms of monitoring range, frequency sensitivity, spatial resolution, and quantitative accuracy. Based on the experimental results, Table 4 presents a comprehensive analysis of the performance of these three sensor types along with supplementary core technical parameters.

DAS provides coverage with a spatial resolution of 1 m, where each signal represents the spatially averaged vibration over a 1 m pipeline segment. FBG sensors are spaced at 50 mm to enable high-precision point strain measurements. Frequency-domain characteristic parameters, such as the 26 Hz blockage characteristic frequency, are scale-invariant and can be directly compared and validated. In contrast, amplitude differences are primarily influenced by the spatial averaging inherent to DAS. High-precision, point-based strain measurements from FBG sensors calibrate DAS fixed-point quantitative errors and spatial-scale discrepancies. Additionally, the high-frequency sensitivity of accelerometers addresses the limitations of DAS in high-frequency monitoring. This multi-scale, multi-principle synergistic approach significantly enhances the accuracy and reliability of pipeline blockage monitoring.

#### 5.4.2. Multi-Sensor Collaborative Monitoring Framework

**Figure 21:**
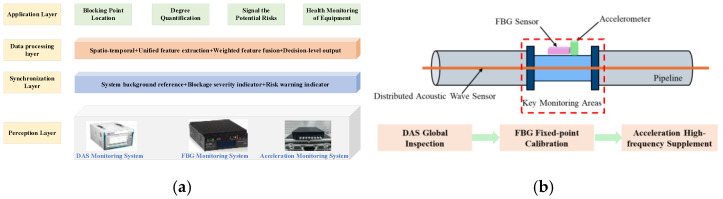
DAS–FBG–accelerometer multi-sensor collaborative monitoring framework: (**a**) system framework diagram; (**b**) schematic diagram of sensor layout.

To address the limitations of single DAS monitoring in fixed-point quantitative accuracy, a “DAS—FBG—accelerometer” multi-sensor collaborative monitoring framework is proposed based on the performance complementarity of the three types of sensors, as shown in Figure 21. This framework, grounded in feature-level data fusion, integrates the advantages of distributed and point-based monitoring, strain and vibration monitoring, as well as low-frequency and high-frequency monitoring, forming a three-dimensional pipeline health perception system of “wide-area screening—precise positioning—detailed diagnosis—quantitative evaluation”, which adapts to the engineering requirements of blockage monitoring for long-distance coal gangue slurry pipelines.

(1) Perception Layer: The DAS fiber functions as the global backbone monitoring network for long-distance pipelines, enabling distributed and continuous monitoring of the entire pipeline. At blockage-prone locations, including valves, bends, and diameter-varying sections, FBG sensors, and accelerometers are deployed to create point-based monitoring nodes, providing high-precision fixed-point monitoring at critical positions. Before experimentation, DAS channels corresponding to the deployment positions of point sensors were localized using the tapping method. These channels are designated as “key synchronous channels” to establish spatial correspondence between distributed DAS data and point-type sensor data.
(2) Synchronization Layer: A unified hardware trigger synchronization scheme was implemented to ensure temporal consistency of multi-source data. All sensors are configured with the same sampling frequency, and a universal synchronous trigger device enables simultaneous data collection from DAS, FBG, and accelerometer systems. This approach establishes the foundation for subsequent feature-level fusion.
(3) Data Processing Layer: Feature-level fusion is conducted through a standardized four-step workflow.
  (1) Spatiotemporal registration: Aligns the DAS, FBG, and accelerometer data using the pre-localized “key synchronous channels” and unified hardware trigger.
  (2) Unified feature extraction produces a common feature vector for each time window, consisting of the amplitudes of three core blockage-correlated frequencies (1.5 Hz, 26 Hz, and 174 Hz energy). These frequency-domain features are inherently scale-invariant, which minimizes sensor-specific amplitude differences.
  (3) Weighted feature fusion linearly combines these features, with weights optimized to leverage the strengths of each sensor: DAS for spatial localization, FBG for quantitative severity assessment, and the accelerometer for transient risk detection.
  (4) Decision-level output inputs the fused feature vector into the sine-fitting quantitative model (R^2^ = 0.985) to generate blockage location, calibrated severity, and risk level.
(4) Application Layer: Based on the analysis results from the data processing layer, four core functions for blockage monitoring of long-distance coal gangue slurry pipelines are achieved: precise localization of blockage points, quantitative evaluation of blockage degree, early real-time warning of transient risks, and health monitoring of equipment status.

This four-layer architecture integrates the advantages of distributed DAS, high-precision FBG, and high-frequency accelerometers, constructing a unified blockage monitoring framework that balances wide-area coverage and localized accuracy.

In this work, multi-sensor data were mainly used for offline cross-validation. The described workflow, featuring low-complexity feature extraction and a sine-fitting model, is designed for real-time implementation. Using standard engineering hardware such as field-programmable gate array (FPGA) acceleration, processing can be completed within seconds, which is suitable for the relatively slow progression of pipeline blockage.

### 5.5. Engineering Application Challenges and Optimization Strategies for Field Monitoring

The distributed acoustic sensing (DAS)-based multi-sensor collaborative monitoring framework developed in this study demonstrated accurate blockage identification, precise localization, and quantitative characterization under controlled laboratory conditions, achieving a goodness of fit of up to 0.985 for the quantitative inversion model. In contrast, the field environment of long-distance coal gangue slurry pipelines in mines is more complex. Several factors challenge the transition from laboratory-scale testing to field engineering application: (1) multi-source industrial noise that may obscure weak blockage signals; (2) environmental influences such as temperature and corrosion that can cause signal drift; (3) long-term degradation of sensing fiber performance; and (4) variable operating conditions, including changes in flow rate and slurry properties, which may alter the resonance window.

To address these core challenges, targeted optimization strategies are proposed that leverage the complementary strengths of the established multi-sensor system. These strategies include the application of advanced signal processing techniques, such as blind source separation, to improve noise immunity; the integration of temperature-compensated FBG nodes to mitigate environmental effects; and the implementation of a two-step calibration method that combines laboratory-based pre-calibration with field in situ adjustment to accommodate variable operating conditions. Collectively, these approaches provide a roadmap for enhancing the engineering robustness and practical applicability of the monitoring framework.

## 6. Conclusions

To address the absence of quantitative methods for coal gangue slurry pipeline blockage monitoring and the limited fixed-point quantification accuracy of standalone distributed acoustic sensing (DAS) technology, this study established a gradient blockage simulation test platform (0–76.42%) integrated with a DAS–FBG–accelerometer multi-source synchronous monitoring system. Systematic experimentation and multi-domain signal analysis yielded the following key findings:

(1) Three core characteristic frequencies (1.5 Hz, 26 Hz, 174 Hz) highly correlated with pipeline blockage were identified and cross-validated using multi-sensor data. These frequencies correspond to system background vibration (grouting pump operation), blockage-induced fluid–structure resonance (local valve–pipe assembly), and transient impact events (slurry collision and cavitation), respectively. The 26 Hz frequency was theoretically verified as the structural natural frequency (Euler–Bernoulli beam model: 25.7 Hz) and cross-validated by DAS, FBG, and accelerometers with a maximum frequency deviation of ≤0.4 Hz, establishing a reliable hierarchical feature perception system that addresses the lack of specific discriminants for slurry pipeline blockage.
(2) The DAS phase change rate exhibited a unimodal nonlinear response to blockage degree, increasing initially and then decreasing, with a peak at 40.94% blockage. Quantitative time-domain analysis showed that the average peak amplitude at 40.94% blockage is 7.25 times that at 8.76% blockage. Based on this, a sine-fitting quantitative inversion model was developed, achieving a high goodness of fit (R^2^ = 0.985). Leave-one-out cross-validation confirmed the model’s robustness, with a mean relative prediction error of 3.77% (<5%), enabling the transition from qualitative blockage detection to quantitative severity assessment.
(3) A feature-level fusion-based collaborative monitoring framework was proposed, leveraging the complementary strengths of DAS, FBG, and accelerometers. This framework integrates the distributed full-pipeline coverage of DAS, the high-precision point quantification of FBG, and the high-frequency transient capture capability of accelerometers. It effectively overcomes the limitations of single DAS monitoring and offers a feasible paradigm for long-distance pipeline health monitoring. In field applications, the framework can achieve four core functions: blockage point localization, quantitative severity evaluation, transient risk early warning, and equipment status monitoring.
(4) Limitations of this study and future research directions are clarified. First, the limited number of experimental blockage gradient groups leads to relatively high parameter uncertainty in the quantitative inversion model. Second, experiments were conducted under controlled laboratory conditions with valve-simulated blockages, while complex field factors such as temperature fluctuations, pipeline corrosion, industrial background vibration, and long-term fiber degradation were not fully addressed. Future research will supplement four to six intermediate blockage gradients to improve the parameter stability and generalization ability of the inversion model and will further focus on natural particle sedimentation blockage experiments and industrial field tests to validate and optimize the proposed framework, thereby enhancing its adaptability to complex field conditions and engineering practicability.

The hierarchical frequency system, quantitative inversion model, and collaborative fusion framework developed in this study provide a robust theoretical and experimental foundation for real-time early warning, precise localization, and intelligent diagnosis of long-distance solid waste slurry pipelines. These advancements are of great significance for improving the safety and efficiency of green backfilling systems in coal mines.

## Figures and Tables

**Table 1:** Pipeline positioning results table for each position.

Pipeline Position	DAS Localization Channel	Pipeline Position	DAS Localization Channel
Pipeline Inlet	15	Rear of Valve 5	79
Front of Valve 2	26	Front of Valve 6	90
Rear of Valve 2	27	Rear of Valve 6	92
Front of Valve 3	49	Front of Valve 7	107
Rear of Valve 3	50	Rear of Valve 7	109
Front of Valve 4	67	Front of Valve 8	123
Rear of Valve 4	69	Rear of Valve 8	124
Front of Valve 5	78	Pipeline Outlet	131

**Table 2:** Average peak amplitude of DAS phase change rate (channels 65–72) under different blockage degrees.

Blockage Degree	0%	8.76%	23.58%	40.94%	59.06%	76.42%
Average Peak Amplitude (a.u.)	0.476	1.905	6.667	13.81	4.286	2.857

**Table 3:** Synchronous measurement results of the 26 Hz characteristic frequency by DAS, FBG, and accelerometer under varying blockage degrees.

Blockage Degree	DAS Measured Frequency (Hz)	FBG Measured Frequency (Hz)	Accelerometer Measured Frequency (Hz)	Maximum Frequency Deviation (Hz)
8.76%	25.7	26.1	25.9	0.4
23.58%	26.1	25.8	26.0	0.3
40.94%	26.0	26.2	25.8	0.4
59.06%	25.9	26.1	25.7	0.4
76.42%	25.8	26.0	25.9	0.2

**Table 4:** Performance comparison and complementarity analysis of DAS, fiber Bragg grating, and accelerometer sensors.

Sensor Type	Monitoring Advantages	Limitations	Complementary Value
DAS	Distributed continuous monitoring, long-distance coverage, strong low-frequency capture capability, capable of locating blockage points	Weak fixed-point quantitative accuracy, lower sensitivity to high-frequency vibration compared with accelerometers	Provides a backbone network for global monitoring and realizes preliminary localization of blockage areas
FBG	Precise point-based strain quantification, strong anti-interference capability, capable of capturing tiny structural deformations	Point-based monitoring with limited range, no distributed localization capability	Calibrates the fixed-point quantitative accuracy of DAS and enables precise calibration of blockage degree
Accelerometer	Sensitive to high-frequency harmonics and transient impacts	Low sensitivity to low-frequency disturbances, susceptible to interference from inherent equipment vibration	Supplements high-frequency vibration information, analyzes vibration source types, and verifies transient impact characteristics

## Data Availability

The original contributions presented in this study are included in the article. Further inquiries can be directed to the corresponding author.
